# Wing loading, not terminal velocity, is the best parameter to predict capacity of diaspores for secondary wind dispersal

**DOI:** 10.1093/jxb/eraa170

**Published:** 2020-04-03

**Authors:** Wei Liang, Zhimin Liu, Minghu Liu, Xuanping Qin, Carol C Baskin, Jerry M Baskin, Zhiming Xin, Zhigang Wang, Zhi Su, Quanlai Zhou

**Affiliations:** 1 Institute of Applied Ecology, Chinese Academy of Sciences, Shenyang, China; 2 University of Chinese Academy of Sciences, Beijing, China; 3 Experimental Center of Desert Forest, Chinese Academy of Forest, Dengkou, China; 4 Department of Biology, University of Kentucky, Lexington, KY, USA; 5 Department of Plant and Soil Sciences, University of Kentucky, Lexington, KY, USA; 6 Royal Holloway, University of London, UK

**Keywords:** Appendage type, diaspore mass, diaspore shape index, downwind slope, terminal velocity, upwind slope, wind tunnel, wing loading

## Abstract

Lift-off velocity may be the most useful surrogate to measure the secondary dispersal capacity of diaspores. However, the most important diaspore attribute determining diaspore lift-off velocity is unclear. Furthermore, it is not known whether terminal velocity used to characterize the primary dispersal capacity of diaspores can also be used to predict their secondary wind dispersal capacity. Here, we investigate how diaspore attributes are related to lift-off velocity. Thirty-six species with diaspores differing in mass, shape index, projected area, wing loading, and terminal velocity were used in a wind tunnel to determine the relationship between diaspore attributes and lift-off velocity. We found that diaspore attributes largely explained the variation in lift-off velocity, and wing loading, not terminal velocity, was the best parameter for predicting lift-off velocity of diaspores during secondary wind dispersal. The relative importance of diaspore attributes in determining lift-off velocity was modified by both upwind and downwind slope directions and type of diaspore appendage. These findings allow us to predict diaspore dispersal behaviors using readily available diaspore functional attributes, and they indicate that wing loading is the best proxy for estimating the capacity for secondary dispersal by wind.

## Teaser

Wing loading is the best proxy for estimating the capacity of diaspores for secondary wind dispersal, which allows us to predict diaspore dispersal behaviors using readily available diaspore functional attributes.

## Introduction

Diaspore dispersal is an important stage in the life history of plants and a crucial mechanism in plant migration and metapopulation dynamics since it determines the colonization process of rare and invasive species (e.g. Poschlod and Bonn, 1998; Kirmer *et al.*, 2008; Hulme, 2009; Lemke *et al.*, 2009). Wind dispersal, including diaspore shedding from the mother plant, primary wind dispersal, and secondary wind dispersal, is one of the most common modes of diaspore dispersal, and it is particularly prevalent in open habitats such as steppes, wastelands, deserts, sand dunes, and road edges (Collins and Uno, 1985; van Rheede van Oudtshoorn and van Rooyen, 1999). Studies have shown that secondary dispersal of diaspores on the ground might move seeds farther from the parent plant than primary dispersal (Watkinson, 1978; Matlack, 1989) and that the final deposition position of wind-dispersed diaspores is determined to a great extent by the process of secondary wind dispersal (Schurr *et al.*, 2005).

Secondary wind dispersal is the movement of diaspores from the time they first land on the ground until they germinate. It can significantly change the seed shadow caused by primary dispersal and may be more important for the spatial distribution pattern of populations and long-distance migration of seeds than primary dispersal (Chambers and MacMahon, 1994; Nathan and Muller-Landau, 2000; Higgins *et al.*, 2003; Schurr *et al.*, 2005). Movement on the ground influences the potential number of diaspores available for germination and establishment at a particular microsite (Harper, 1977). The likelihood of diaspore movement is a function of morphological traits, configuration of the ground surface, and wind velocity (Harper *et al.*, 1965, 1970; Sheldon, 1974; Chambers *et al.*, 1991; Johnson and Fryer, 1992). Although researchers have attempted to evaluate the secondary dispersal capacity of diaspores (Matlack, 1989; Greene and Johnson, 1997; Wall and Joyner, 1998; Schurr *et al.*, 2005), empirical studies are scarce, and the range of species and impact indicators involved are far from comprehensive.

Lift-off velocity (or threshold velocity) is the wind velocity that causes a diaspore to move (Arnold and Weihs, 1978), and it can be used to represent diaspore movements over surfaces when studying secondary dispersal by wind (Schurr *et al.*, 2005). Consequently, lift-off velocity may be the most useful surrogate to measure secondary dispersal capacity of diaspores. Although studies have confirmed that the lift-off velocity differs with various types of diaspores (Lemke *et al.*, 2009), how modifications of diaspore traits influence lift-off velocity has not been explored. Controlled experiments on natural topography in the field are a big challenge for studies of secondary dispersal of diaspores by wind. Existing mechanistic models of secondary diaspore dispersal by wind usually lack experimental validation, and the relatively small number of species involved in empirical research may be insufficient to reflect general trends.

The topography in nature is usually undulating, and slope direction can affect the process of diaspore dispersal (Li, 2008; Liang *et al.*, 2019). However, whether topographic undulation will change the relative contribution of various diaspore attributes to the variation in lift-off velocity remains unknown. Thus, it is essential to determine if the effect of diaspore attributes on lift-off velocity is modified by slope direction.

Species may differ in their dispersal abilities because of differences in the stability of the habitats they occupy (Gadgil, 1971) and/or because they have different diaspore attributes. Diaspores with obvious morphological adaptations for wind dispersal vary enormously in effectiveness of dispersal (Sheldon and Burrows, 1973). Diaspore characteristics such as size, shape, mass, appendage type, and wing loading are reported to affect dispersal (Burrows, 1973; Green, 1980; Guries and Nordheim, 1984; Andersen, 1992; Thompson *et al.*, 1993; Funes *et al.*, 1999). Some diaspore appendages such as bristles or thorns may be trapped in crevices, thus inhibiting secondary dispersal by wind. Species lacking obvious structural modifications might achieve considerable wind dispersal by virtue of small diaspore size or spherical shape. Projected area can affect the ability of diaspores to be lifted by wind. Wing loading, reflecting air resistance during the flight, is a frequently used parameter in studying wind dispersal of diaspores (e.g. Green, 1980; Andersen, 1993; Schurr *et al.*, 2005; Lentink *et al.*, 2009). However, we do not know whether the decisive role of a diaspore attribute originally considered important will change when additional attributes (such as mass, shape, projected area, and wing loading) are considered at the same time. A comparison of multiple species with various diaspore attributes in different terrains may lead to more general conclusions. Since movement of diaspores along the ground must overcome ground friction caused by the difference between seed gravity and the lift developed (Johnson and Fryer, 1992), we hypothesize that lift-off velocity is determined by diaspore mass.

Terminal velocity is defined as a steady and maximum speed when air resistance is equal to the pull of gravity during a free fall of seeds in motionless air (Green, 1980). It is considered as the most useful indirect parameter of primary dispersal capacity in diaspore wind dispersal models, because diaspores with a low terminal velocity may fly longer in the air and thus disperse a longer distance than diaspores with a high terminal velocity (Okubo and Levin, 1989; Greene and Johnson, 1990; Caplat *et al.*, 2012). However, it is not known if the terminal velocity can replace wing loading in characterizing lift-off velocity of diaspores. Moreover, whether the terminal velocity used to characterize primary seed dispersal capacity also can be used to predict secondary dispersal capacity of the seeds is well worth exploring. Hence, we hypothesize that good positive correlations exist between lift-off velocity and terminal velocity, and that terminal velocity can be used as an indicator for assessing wind dispersal capacity of diaspores.

In this study, 36 species with different diaspore attributes (appendage type, mass, projected area, shape index, wing loading, and terminal velocity) were evaluated in the field on upwind and downwind slopes to determine how lift-off velocity is related to diaspore attributes and slope direction during the secondary diaspore dispersal by wind. We asked two questions. (i) Which diaspore attribute is the most important in determining lift-off velocity? (ii) Is terminal velocity a decisive factor for determining lift-off velocity?

## Materials and methods

### Diaspore selection

Diaspores were collected from the meadow grassland, steppe, desert, sand dune, and riparian woodland in Inner Mongolia from July 2017 to August 2018, and then air-dried naturally in the laboratory for >2 weeks. Differences in traits of the selected species were not restricted by phylogeny, and each diaspore was only considered as a representation of its own morphological attributes. Diaspores with various kinds of appendages and a gradient of shapes and sizes were included in the experiment. Air-dried diaspores of 36 species, with (wing, thorn, or hair) or without appendages, a mass range of 1.12–231.46 mg, and a shape index range of 0.001–0.194 (Table 1) were selected to conduct lift-off velocity measurements. Among the 36 species, nine did not have an appendage, seven had hairs, six had thorns, and 14 had wings.

**Table 1.** Diaspore attributes relevant to wind dispersal of the 36 studied species (mean ±SE)

Species	Appendage type	Mass (mg)	Projected area (mm^2^)	Shape index	Wing loading (mg mm^−2^)	Terminal velocity (m s^−1^)
***Heracleum dissectum***	Wing	4.770±0.603	38.527±2.683	0.148±0.005	0.124±0.012	1.544±0.184
***Haloxylon ammodendron***		6.776±1.383	36.839±4.368	0.063±0.022	0.186±0.040	1.685±0.353
***Syringa oblata***		9.905±1.807	25.481±2.892	0.152±0.011	0.394±0.086	2.428±0.270
***Althaea rosea***		16.935±0.613	44.744±1.626	0.119±0.013	0.379±0.020	2.728±0.473
***Ailanthus altissima***		17.090±1.754	191.062±11.037	0.173±0.003	0.090±0.010	1.043±0.060
***Ferula bungeana***		21.225±2.766	50.520±8.331	0.129±0.008	0.428±0.075	2.740±0.223
***Atriplex canescens***		31.350±8.569	77.276±13.293	0.007±0.005	0.409±0.093	2.485±0.228
***Acer negundo***		35.799±5.255	197.827±20.144	0.160±0.002	0.181±0.015	0.780±0.066
***Calligonum leucocladum***		52.095±4.929	166.598±16.548	0.002±0.003	0.314±0.028	2.255±0.164
***Zygophyllum xanthoxylon* (disc)**		90.685±18.334	603.932±83.549	0.142±0.016	0.152±0.031	1.646±0.213
***Calligonum rubicundum***		149.975±25.795	218.669±26.680	0.003±0.003	0.686±0.082	3.098±0.141
***Zygophyllum xanthoxylon* (four-winged)**		163.470±43.580	558.871±102.138	0.013±0.007	0.291±0.045	1.959±0.244
***Koelreuteria paniculata***		178.635±20.052	765.118±85.573	0.167±0.004	0.237±0.043	2.475±0.385
***Acer nikoense***		231.460±26.136	491.381±33.327	0.136±0.002	0.471±0.058	1.103±0.082
***Tragus berteronianus***	Thorn	1.065±0.496	5.125±0.446	0.067±0.023	0.211±0.105	1.844±0.416
***Lappula redowskii***		6.775±1.664	11.156±1.702	0.004±0.003	0.607±0.120	2.225±0.441
***Agrimonia pilosa***		16.155±2.413	15.584±2.210	0.056±0.006	1.039±0.074	3.090±0.266
***Tribulus terrestris***		26.345±13.790	24.244±5.742	0.016±0.008	1.167±0.740	2.714±0.239
***Calligonum alaschanicum***		44.420±10.625	73.215±16.064	0.007±0.004	0.615±0.122	3.285±0.116
***Xanthium sibiricum***		74.660±21.572	45.319±3.998	0.027±0.004	1.677±0.576	3.729±0.213
***Aster tataricus***	Hair	1.120±0.233	24.731±5.720	0.057±0.021	0.046±0.010	0.622±0.111
***Cirsium japonicum***		2.340±1.109	203.032±43.006	0.001±0.002	0.013±0.008	0.315±0.045
***Syneilesis aconitifolia***		4.660±1.146	50.910±14.036	0.027±0.010	0.097±0.033	1.305±0.380
***Catalpa ovata***		5.005±1.227	73.216±16.911	0.194±0.004	0.069±0.015	1.078±0.173
***Atractylodes coreana***		6.865±1.841	35.125±7.469	0.007±0.005	0.205±0.075	0.749±0.112
***Echinops gmelini***		9.115±1.860	40.598±4.212	0.006±0.004	0.227±0.055	2.195±0.225
***Atractylodes japonica***		11.625±4.031	34.085±5.061	0.041±0.024	0.351±0.136	2.493±0.194
***Viola yezoensis***	None	1.115±0.239	1.388±0.160	0.031±0.009	0.819±0.231	0.551±0.031
***Scirpus planiculmis***		2.320±0.221	5.668±0.425	0.092±0.004	0.410±0.039	0.660±0.044
***Kummerowia striata***		2.393±0.345	4.303±0.367	0.088±0.020	0.558±0.084	1.171±0.094
***Carex lehmanii***		5.165±0.403	8.218±0.635	0.101±0.005	0.631±0.057	1.258±0.461
***Panicum bisulcatum***		5.125±0.693	4.705±0.492	0.046±0.006	1.103±0.200	1.792±0.190
***Thermopsis lanceolata***		18.185±3.573	9.322±0.781	0.019±0.003	1.956±0.378	2.858±0.371
***Platycladus orientalis***		22.405±10.257	14.758±1.586	0.062±0.011	1.537±0.726	3.010±0.390
***Euonymus bungeanus***		40.865±10.544	20.209±2.554	0.041±0.009	2.037±0.526	2.470±0.252
***Messerschmidia sibirica***		66.600±7.452	34.095±3.187	0.010±0.006	1.961±0.234	3.457±0.262

For each type of appendage, species are listed in order of increased mass.

The disc-shaped diaspore is shaped like ‘–’, with its two wings nearly on the same horizontal plane; the four-winged diaspore is shaped like ‘+’, with the adjacent wings at angles of 90°

### Traits measurement

Diaspore attributes, including mass, shape index, wing loading, projected area, and terminal velocity, were selected to ascertain the determinants of diaspore lift-off velocity, since they frequently have been used for measuring wind dispersal of diaspores (Casper and Grant, 1988; Matlack, 1992; Casseau *et al.*, 2015). Twenty intact diaspores of each species were selected for the measurements.

Length, width, and height of diaspores were measured with Vernier calipers (0.01 mm accuracy). The shape index (*V*_s_) was calculated as in Thompson *et al.* (1993):

$$\begin{array}{l} V_{s}=\frac{\overset{}{\sum }{\left[x_{i}-\frac{\overset{}{\sum }x_{i}}{3}\right]}^{2}}{N} \end{array}$$

Where *N*=3, $x_{1}=\frac{Length}{Length}=1$, $x_{2}=\frac{Width}{Length\;\;\;}$, and $x_{3}=\frac{Height}{Length}$. Diaspore mass was measured using an electronic balance (0.1 mg accuracy). After scanning with a digital scanner, the projected area of each diaspore was measured with image analysis software Motic Image Plus 2.0 (Motic China Group Co., Ltd, USA). Wing loading (ɷ)was calculated as in Matlack (1987):

$$\begin{array}{l} \omega =\frac{m}{p} \end{array}$$

where *m* is the diaspore mass and *p* is the projected area. The terminal velocity was defined as the constant falling velocity of a diaspore in still air (Green, 1980), and it was measured with an apparatus described by Zotz *et al.* (2016).

### The wind tunnel

**Fig. 1.**
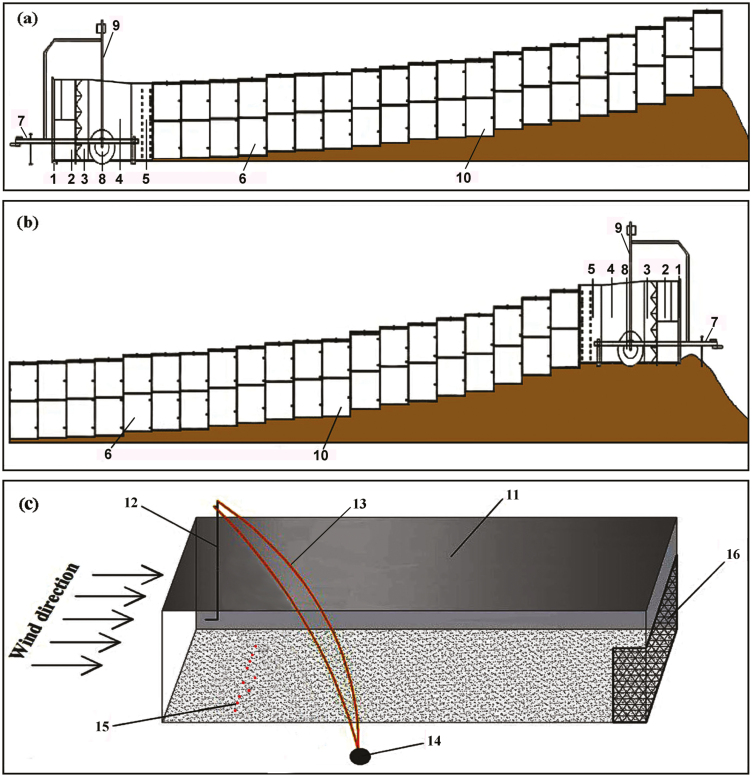
Diagram illustrating measurement of the diaspore lift-off velocity by wind tunnel. (1) Starting point section, (2) power section, (3 and 4) diversion section, (5) rectifying section, (6) transition section, (7) draft gear, (8) road wheel, (9) electric landing gear, (10) experimental section, (11) experimental section of wind tunnel, (12) pitot tube, (13) rubber tube, (14) differential pressure transmitter, (15) diaspore, (16) wire net. The wind tunnel is 2 m in height, 2 m in width, and 20m in length. The upwind slope is 6–8º with the power section of wind tunnel at the lower part of the slope (a). The downwind slope is 6–8º with the power section at the upper part of the slope (b). The pitot tube was located 10 m away from the power section. It was inserted through the roof via the pitot hole, and wind speed was measured 1 m above the underlying surface (c).

The wind tunnel was 2 m high and 2 m wide, with a 20 m long test section consisting of twenty 1 m segments that could be adjusted to conform to the soil surface (Fig. 1). The standard component of the wind tunnel experimental section was 1 m×2 m and could be installed on an undulating surface. Wind speed was monitored inside the tunnel using a pitot tube (160-96, Dwyer Instruments, Inc., IN, USA) connected to a Magnesense II Differential Pressure Transmitter (MS2-W102-LCD, Dwyer Instruments, Inc.). The pitot tube was located 10 m away from the power section. It was inserted through the roof via the pitot hole, and wind speed was measured 1 m above the underlying surface. The use of a large-scaled, flexible portable field wind tunnel made it possible to conduct controlled experiments on undulating terrain (Liang *et al.*, 2019).

### The underlying surface matrix

The aeolian sand from a moving sand dune was chosen as the underlying surface matrix. In this study, a slope with a gradient of ~6° and 50 m in length was selected in the Ulanbuh sand dune field in Inner Mongolia, China (106°35'E, 40°17'N) as the site on which to conduct the wind tunnel experiments. The surface matrix (sand) was naturally air-dried and then sieved with a mesh size of 5 mm to remove non-substrate impurities (such as stones and pieces of plants). The experimental section was then manually made smooth and straight.

### Measurement of lift-off velocity

Thirty-six species were used to conduct the experiments on the upwind slope and downwind slope (Liang *et al.*, 2019). When the experiment was conducted on the upwind slope, the power section of the wind tunnel was at the lower part of the slope; that is, the wind blew up the slope (Fig. 1a). When the experiment was conducted on the downwind slope, the power section was at the upper part of the slope; that is, the wind blew down the slope (Fig. 1b).

The experimental section was set 10 m from the air inlet. Ten diaspores were released simultaneously from a height of 10 cm above the dry sand surface to ensure randomness of the initial orientation of the diaspores. Then, the fan motor frequency was increased from 0 in increments of 0.01 Hz until at least two diaspores were moved, which would be defined as the lift-off velocity of the diaspores (Johnson and Fryer, 1992). As the fan motor power was increased, wind speed at a height of 1 m from the underlying surface was recorded. The experiment was repeated five times for each species (Fig. 1c).

### Data analysis

Ordination analysis was conducted to assess variation of lift-off velocity corresponding to explanatory factors (diaspore shape index, mass, projected area, wing loading, and terminal velocity). Canonical correspondence analyses (CCAs) based on correlation matrixes of lift-off velocity and explanatory factors were conducted using Canoco 5.0 (Microcomputer Power, Ithaca, NY, USA) (Tackenberg, 2003). The contribution of diaspore attributes to the variation in lift-off velocity was tested following standardized interactive-forward-selection procedures. Statistical analyses were conducted using IBM SPSS Statistics 22.0 (IBM Corporation 1989 and 2013, USA). One-way ANOVA was used to analyze changes in lift-off velocity of each species dispersed on upwind and downwind slopes. Pearson correlation analysis was conducted to analyze the relationship between lift-off velocity and measured diaspore attributes. Regression analysis was only used to analyze the relationship between lift-off velocities and correlated morphological parameters. Linear mixed model analysis was conducted to test the interaction effect of wing loading (as the fixed factor) and appendage type (as the random factor) on the lift-off velocity.

## Results

### Contribution of diaspore attributes to lift-off velocity

The lift-off velocity of diaspores on the upwind slope was higher than that on the downwind slope (Table 2). Based on CCAs, the total explanation of the diaspore attributes on lift-off velocity on the upwind and downwind slopes was 79.6% and 78.5%, respectively. For all species, lift-off velocity on the upwind slope was positively correlated with wing loading (explained 77.3% of the variation in lift-off velocity) and terminal velocity (explained 22.1% of the variation), while, on the downwind slope, it was positively correlated only with wing loading (explained 76.0% of the variation) (Tables 3, 6).

**Table 2.** Lift-off velocity of the 36 studied species on the upwind and downwind slope (mean ±SE)

Species	Appendage type	Lift-off velocity (m s^−1^)		Significant difference
		Upwind slope	Downwind slope	
***Heracleum dissectum***	Wing	5.278±0.440	4.050±0.281	***
***Haloxylon ammodendron***		4.197±0.338	3.585±0.150	**
***Syringa oblata***		7.023±0.717	5.962±0.469	*
***Althaea rosea***		6.914±0.343	5.964±0.352	**
***Ailanthus altissima***		5.087±0.612	3.891±0.191	**
***Ferula bungeana***		6.222±0.660	5.211±0.227	*
***Atriplex canescens***		5.537±0.167	3.470±0.131	***
***Acer negundo***		8.797±0.207	5.294±1.044	***
***Calligonum leucocladum***		4.876±0.316	3.715±0.127	***
***Zygophyllum xanthoxylon* (disc)**		4.892±0.299	3.764±0.204	***
***Calligonum rubicundum***		5.937±0.495	3.878±0.529	***
***Zygophyllum xanthoxylon* (four-winged)**		4.443±0.229	3.523±0.253	***
***Koelreuteria paniculata***		4.955±0.408	3.701±0.248	***
***Acer nikoense***		11.069±0.662	10.109±1.155	NS
***Tragus berteronianus***	Thorn	5.332±0.174	4.268±0.368	***
***Lappula redowskii***		5.887±0.964	4.944±0.148	NS
***Agrimonia pilosa***		6.822±0.180	5.482±0.303	***
***Tribulus terrestris***		8.809±0.463	6.368±0.312	***
***Calligonum alaschanicum***		5.774±0.230	4.273±0.541	***
***Xanthium sibiricum***		11.284±0.816	10.436±0.634	NS
***Aster tataricus***	Hair	2.582±0.004	2.517±0.093	NS
***Cirsium japonicum***		2.108±0.002	2.306±0.130	**
***Syneilesis aconitifolia***		3.125±0.344	2.610±0.214	*
***Catalpa ovata***		4.195±0.298	3.622±0.116	**
***Atractylodes coreana***		3.192±0.193	2.624±0.183	**
***Echinops gmelini***		4.416±0.284	3.151±0.116	***
***Atractylodes japonica***		3.689±0.467	3.292±0.228	NS
***Viola yezoensis***	None	5.617±0.731	5.540±0.440	NS
***Scirpus planiculmis***		7.104±0.525	6.447±0.214	*
***Kummerowia striata***		5.572±0.783	7.422±0.177	NS
***Carex lehmanii***		5.107±0.426	5.016±0.529	NS
***Panicum bisulcatum***		9.891±0.377	9.550±0.278	NS
***Thermopsis lanceolata***		10.659±0.661	9.227±0.415	**
***Platycladus orientalis***		7.577±0.947	6.654±0.541	NS
***Euonymus bungeanus***		12.324±0.217	11.333±0.340	***
***Messerschmidia sibirica***		9.462±1.235	6.987±0.790	**

For each type of appendage, species are listed in order of increased mass.

The disc-shaped diaspore is shaped like ‘–’, with its two wings nearly on the same horizontal plane; the four-winged diaspore is shaped like ‘+’, with the adjacent wings at angles of 90°. *0.011<*P*<0.05, **0.0011<*P*<0.01, *** *P*<0.001, NS, not significant.

**Table 3.** Percentage of total variation of lift-off velocity on the upwind slope and downwind slope explained by wing loading, terminal velocity, diaspore mass, projected area, and shape index

Diaspore attributes	Upwind slope			Downwind slope		
	Explanation %	*F*	*P*	Explanation %	*F*	*P*
**Wing loading**	77.3	54.4	0.001**	76.0	50.3	0.001**
**Terminal velocity**	22.1	7.2	0.014*	12.6	3.7	0.060
**Diaspore mass**	7.1	2.0	0.164	3.7	1.0	0.328
**Projected area**	1.5	0.4	0.551	3.2	0.9	0.387
**Shape index**	<0.1	<0.1	0.883	<0.1	<0.1	0.909
**Total**	79.6	–	–	78.5	–	–

The total explanation of the diaspore attributes on variation of lift-off velocity was calculated by the canonical correlation analysis.

*0.011<*P*<0.05, **P<0.01

The lift-off velocity was positively correlated with wing loading for the diaspores with thorns or without appendages on the upwind slope, while it was positively correlated with wing loading only for diaspores with thorns on the downwind slope. In addition to wing loading, for both slope directions, lift-off velocity was positively correlated with diaspore mass and terminal velocity for diaspores with appendages (thorns, wings, or hairs) and with diaspore mass and shape index for diaspores with wings or hairs (Table 6). The linear mixed model analysis showed that the lift-off velocity of diaspores is affected by the interaction effect of their wing loading and appendage type (*P*=0.001).

### Responses of lift-off velocity to wing loading

**Fig. 2.**
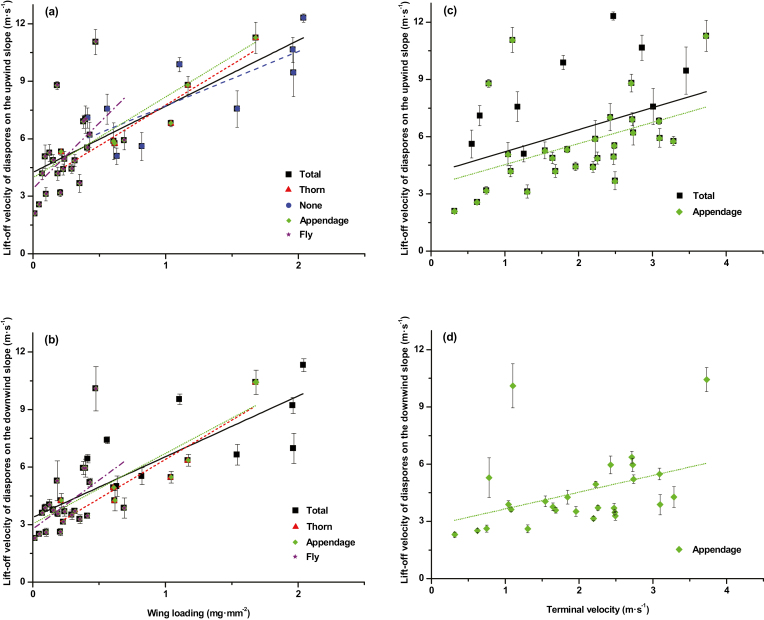
Relationship between lift-off velocities on upwind (a, c) and downwind (b, d) slopes, and wing loading and terminal velocity for diaspores with different types of appendage. Values shown were mean ±SE. Total, Thorn, None, Appendage, and Fly, diaspores of all 36 species, diaspores with thorns, diaspores without appendages, diaspores with any appendages (wings, thorns, or hairs), and diaspores with wings or hairs, respectively. Solid regression lines correspond to 36 species (squares), short-dashed lines to diaspores with thorns (triangles), long-dashed lines to diaspores without any appendages (circles), short-dotted lines to diaspores with appendages (rhombi), and dash–dot lines to diaspores with appendages (pentagrams).

Wing loading was the most important factor determining lift-off velocity of the diaspores (Table 3). On the upwind slope, lift-off velocity of total diaspores, diaspores with thorns, diaspores without an appendage, diaspores with appendages (wings, hairs, or thorns), and diaspores with wings or hairs increased with increasing wing loading (Fig. 2a). The slope of the regression equation was ordered as ‘Fly’>‘Thorn’>‘Appendage’>‘Total’>‘None’ (Table 4). However, on the downwind slope, positive linear relations existed only between lift-off velocity and wing loading for total diaspores, thorned diaspores, diaspores with appendages (wings, hairs, or thorns), and diaspores with wings or hairs (Fig. 2b). The slope of the regression equation was ordered as ‘Fly’>‘Thorn’>‘Appendage’>‘Total’ (Table 4).

**Table 4.** Significant linear regression between lift-off velocity and wing loading of diaspores with different appendage types on upwind and downwind slopes

Appendage type	Upwind slope			Downwind slope		
	Regression equation	*R* ^2^	*P*	Regression equation	*R* ^2^	*P*
**Fly**	*y*=6.8405*x*+3.4259	0.3081	0.009	*y*=5.1047*x*+2.8064	0.2492	0.021
**Thorn**	*y*=4.2228*x*+3.5777	0.8963	0.004	*y*=4.0925*x*+2.3368	0.8232	0.012
**Appendage**	*y*=4.2131*x*+3.9820	0.4980	0.000	*y*=3.6748*x*+3.0675	0.4960	0.000
**Total**	*y*=3.4491*x*+4.2497	0.6153	0.000	*y*=3.1658*x*+3.3768	0.5968	0.000
**None**	*y*=2.8418*x*+4.8908	0.6171	0.012	–	–	–

Total, diaspores of all species; Appendage, diaspores with any appendages (wings, thorn, or hairs); Fly, diaspores with wings or hairs; Thorn, diaspores with thorns; None, diaspores without any appendages.

### Responses of lift-off velocity to terminal velocity

Terminal velocity was another relevant factor that determined the lift-off velocity of diaspores (Table 3). Terminal velocity was proportional to lift-off velocity of total diaspores and diaspores with appendages (wings, hairs, or thorns) on the upwind slope (Fig. 2c). However, on the downwind slope, it was positively related only to diaspores with appendages (wings, hairs, or thorns) (Fig. 2d). The slope of the regression equation was ordered as ‘Total–Upwind’>‘Appendage–Upwind’>‘Appendage–Downwind’ (Table 5).

**Table 5.** Significant linear regression between lift-off velocity and terminal velocity of diaspores with different appendage types on upwind and downwind slopes

Appendage type	Upwind slope			Downwind slope		
	Regression equation	*R* ^2^	*P*	Regression equation	*R* ^2^	*P*
**Appendage**	*y*=1.1040*x*+3.4541	0.1756	0.023	*y*=0.8773*x*+2.7771	0.1576	0.040
**Total**	*y*=1.1551*x*+4.0538	0.1756	0.011	–	–	–
**None**	*y*=2.8418*x*+4.8908	0.6171	0.012	–	–	–

Total, diaspores of all species; Appendage, diaspores with any appendages (wings, thorn, or hairs); None, diaspores without any appendages

## Discussion

Determination of the parameters that can reasonably characterize the capacity of diaspores for secondary dispersal by wind is an important focus of dispersal biology (Harper, 1977; Watkinson, 1978; Johnson and Fryer, 1992; Redbo-Torstensson and Telenius, 1995). Our study indicates that diaspore attributes largely explain the variation in lift-off velocity, and wing loading is the most important one. Terminal velocity is not sufficient to characterize the capacity of diaspores for secondary dispersal by wind because its correlation with lift-off velocity is modified by slope direction and diaspore appendages. The decisive role of a morphological indicator might change when more diaspore traits are included in the comparison (Greene and Johnson, 1997). In our study, diaspore attributes could largely explain the variation in lift-off velocity for either slope direction. However, the significant factors influencing lift-off velocity varied with slope direction. Wing loading was always the most important factor affecting lift-off velocity regardless of slope direction, while terminal velocity played an important role only on the upwind slope (Table 3). The terminal velocity only becomes significant when seed movement requires a large upward lifting force (on the windward slope in this study).

Wing loading might be an important parameter for predicting lift-off velocity. Johnson and Fryer (1992) showed that the square root of wing loading was closely associated with lift-off velocity of some conifer species, but this conclusion was not applicable when additional species were taken into consideration (Greene and Johnson, 1997). Here, we paid attention to the relative role of wing loading in determining lift-off velocity and confirmed that among many diaspore attributes wing loading was indeed the most pivotal parameter influencing lift-off ability. However, the influence of wing loading on lift-off varied with the kind of diaspore appendages (Table 6). Diaspore appendages may affect lift-off velocity by direct trypanospermy (restriction of dispersal due to an anchorage mechanism) or indirect regulation of wing loading (van Rheede van Oudtshoorn and van Rooyen, 1999).

**Table 6.** Correlation matrix between diaspore attributes and lift-off velocity parameters

Lift-off velocity	Diaspore mass		Projected area		Shape index		Wing loading		Terminal velocity	
	*r*	Significance	*r*	Significance	*r*	Significance	*r*	Significance	*r*	Significance
**Up**	0.237	0.164	–0.110	0.523	0.026	0.879	0.784**	0.000	0.419*	0.011
**Down**	0.170	0.320	–0.159	0.354	0.021	0.905	0.772**	0.000	0.314	0.062
**Up–down**	0.262	0.123	0.140	0.414	0.007	0.966	0.208	0.224	0.410*	0.013
**Wing (up)**	0.298	0.300	–0.004	0.989	0.344	0.228	0.348	0.222	–0.316	0.270
**Thorn (up)**	0.781	0.067	0.232	0.658	–0.144	0.786	0.947**	0.004	0.670	0.146
**Hair (up)**	0.695	0.083	–0.519	0.233	0.407	0.365	0.553	0.198	0.734	0.060
**None (up)**	0.566	0.112	0.473	0.198	–0.571	0.108	0.786*	0.012	0.638	0.064
**Wing (down)**	0.337	0.238	0.028	0.924	0.363	0.202	0.339	0.235	–0.250	0.389
**Thorn (down)**	0.792	0.060	0.205	0.697	–0.084	0.874	0.907*	0.012	0.660	0.154
**Hair (down)**	0.601	0.153	–0.368	0.417	0.682	0.091	0.458	0.302	0.644	0.118
**None (down)**	0.313	0.412	0.213	0.582	–0.408	0.275	0.614	0.079	0.423	0.257
**Appendage (up)**	0.392*	0.043	0.052	0.796	0.205	0.305	0.706**	0.000	0.437*	0.023
**Appendage (down)**	0.384*	0.048	0.034	0.868	0.195	0.331	0.704**	0.000	0.397*	0.040
**Fly (up)**	0.495*	0.023	0.233	0.308	0.476*	0.029	0.555**	0.009	0.210	0.362
**Fly (down)**	0.493*	0.023	0.218	0.343	0.479*	0.028	0.499*	0.021	0.136	0.557

Up, lift-off velocity of diaspore on the upwind slope; Down, lift-off velocity of diaspore on the downwind slope; Up–down, difference between lift-off velocity on the upwind and downwind slope; Appendage, diaspores with any appendages (wings, thorn, or hairs); Fly, diaspores with wings or hairs; Wing, Thorn, Hair; and None, diaspores with wings, thorns, hairs, and without any appendages, respectively. *0.011<*P*<0.05, **P<0.01

Anemochorous diaspores usually have appendages such as wings, hairs, balloons, or thorns (Augspurger, 1986; Matlack, 1987). In our study, lift-off velocity for diaspores with thorns was positively correlated with wing loading (but not with other diaspore attributes) for both slope directions, but it was correlated with wing loading for diaspore with hairs or wings (as well as other diaspore attributes) for both slope directions. Diaspores without appendages, in which lift-off velocity was related only to wing loading on the upwind slope, were more difficult to move by wind than diaspores with the other three types of appendages (Tables 2, 6). Appendage type and slope direction together determined the difference in diaspore attributes critically related to lift-off velocity. Diaspores without wind-dispersal structures, even if they are relatively light and small, often require higher wind speeds to move than those with these structures (Maddox and Carlquist, 1985). The location of the center of the mass of the diaspore also may cause differences in their capacity for secondary dispersal by wind (van Rheede van Oudtshoorn and van Rooyen, 1999). A thorn is capable of promoting horizontal movement of diaspores by supporting the center of mass above the ground and increasing diaspore area in contact with the wind, which explains why the lift-off velocity of thorned diaspores varied most with wing loading.

Diaspore mass is an important factor in determining secondary dispersal by wind, and Greene and Johnson (1997) suggested that lift-off velocity decreased with diaspore mass. However, our results show that diaspore mass had significant effects only on lift-off ability of diaspores with appendages, especially those that may facilitate flight (Table 6). This result is quite different from our hypothesis that the lift-off velocity is critically determined by diaspore mass. The effect of gravity may be relatively small for tumbling seeds.

Shape also may be a factor affecting wind dispersal capacity of diaspores (Casseau, 2015; Planchuelo *et al.*, 2016). Presumably, spherical diaspores are more likely to start tumbling on the ground than elongated ones. However, our results showed that only diaspores with wings or hairs showed a significant positive correlation between shape index and lift-off velocity (Table 6). Thus, diaspore shape is an important indicator of secondary wind dispersal for diaspores with appendages that are conducive to flight (such as wings or hairs).

The comparison of multiple species with various diaspore attributes on up and down slopes gives significant insight into how diaspore morphology affects the capacity of diaspores for secondary dispersal by wind. By combining topographic factors with diaspore morphology, we found that wing loading is the best factor to characterize the capacity of diaspores for secondary dispersal by wind. This result provides theoretical support for the establishment of a model for secondary dispersal by wind and for evaluation of the capacity of diaspores to be wind dispersed.

Terminal velocity might be an important indirect parameter for predicting the capacity of diaspores for secondary dispersal by wind, since it is thought to be linked to dispersal distance (Andersen, 1992; Tackenberg, 2003; Soons *et al.*, 2004; Nathan *et al.*, 2011; Caplat *et al.*, 2012; Zotz *et al.*, 2016). In our study, terminal velocity was correlated to lift-off velocity for all species only on the upwind slope and in species with diaspore appendages on both slope directions.

As mentioned above, although the terminal velocity was correlated with the lift-off velocity in some cases and could explain the variance in lift-off velocity between upwind and downwind slopes, its explanatory power for lift-off velocity of secondary dispersal was much lower than that for wing loading. Therefore, contrary to our hypothesis, terminal velocity is not the best indicator for assessing the capacity of diaspores for secondary dispersal by wind, although it can be used to estimate the difference between the lift-off velocity of the same diaspore on the upwind and downwind slopes.

Previous studies have suggested that the terminal velocity of diaspores is the most important variable in predicting dispersal distance by wind (e.g. Nathan *et al.*, 2011; Tamme *et al.*, 2014; Zhu *et al.*, 2015; Smith *et al.*, 2016), However, our study shows that it is not the best parameter to predict capacity of diaspores for secondary wind dispersal. Therefore, we suggest that terminal velocity is the best factor for assessing the capacity of diaspores for primary dispersal by wind, and wing loading is the best factor for assessing the capacity of diaspores for secondary dispersal by wind.

In conclusion, wing loading is a better attribute to characterize the capacity of diaspores for secondary dispersal by wind than terminal velocity. The effect of diaspore morphology on lift-off ability during secondary dispersal by wind varies with the kind of diaspore appendage and slope direction: up or down. Our results suggest that by calculating wing loading of the diaspores, the wind velocity at which diaspores will start to move can be predicted and thus the wind velocity below which they will remain stationary and germinate when temperature and soil moisture is sufficient for them to do so. Estimation of diaspore dispersal capacity can facilitate a more rational screening of the species to use for management and restoration of degraded habitats.

## Author contributions

ZL, WL, ML, ZW, and ZS conceived the ideas and designed the methodology. WL, XQ, QZ, and ZX collected the data. WL analyzed the data and led the writing of the first draft of the manuscript. ZL, CCB, and JMB revised several drafts of the manuscript. All authors gave final approval for publication.
